# Model Structures and Electron Transfer Properties of Conductive Nickel-Organic Nanoribbons in Cable Bacteria

**DOI:** 10.1021/acs.jpclett.6c01926

**Published:** 2026-08-31

**Authors:** Oliver Russell, Martijn A. Zwijnenburg, Filip J.R. Meysman, Jochen Blumberger

**Affiliations:** † Department of Physics and Astronomy, 4919 University College London, London WC1E 6BT, United Kingdom; ‡ Thomas Young Centre, London WC1E 6BT, United Kingdom; ¶ Department of Chemistry, London WC1H 0AJ, United Kingdom; § Department of Biology, 26660 University of Antwerp, Antwerp 2610, Belgium

## Abstract

Cable bacteria are multicellular bacteria capable of centimeter-scale conduction through a regular fiber network embedded in their cell envelope. The conductivity of these fibers is extremely high for biological materials and rivals that of the best synthetic conductive polymers, but the underlying electron transport mechanism remains elusive. Recent microscopic and spectroscopic evidence indicates that each fiber embeds a bundle of intertwined nanoribbons as the conductive conduit. Each nanoribbon consists of a one-dimensional nickel-organic framework, built from stacked nickel bis­(1,2-dithiolene) oligomers (NiBiD units) as molecular building blocks. Here we performed DFT calculations of nanoribbon model structures, in order to characterize their electronic properties, examine potential stacking configurations and verify whether these structures can support efficient conductance. Our simulations indicate that nanoribbons are composed of tightly stacked AA or AB-type packings of NiBiD units. In the most energetically stable structure (AB-type) some Ni centers are predicted to be 5-fold coordinated due to formation of an interlayer Ni–S coordination bond. In several energetically low-lying structures, the electronic coupling between neighboring molecules exceeds the critical threshold for charge delocalization permitting efficient charge transport beyond small polaron hopping. Our results hence reveal that nanoribbons based on NiBiD units exhibit favorable charge transfer properties that may explain the unusually high conductivities measured in the fibers of cable bacteria.

## Graphical abstract

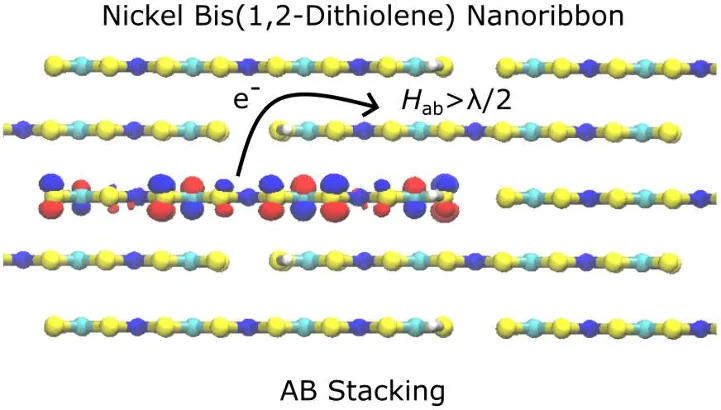

Electron transfer (ET) is at the heart of virtually all energy conversion processes in living organisms, with photosynthesis and respiration providing the most prominent examples.^1,2^ At the cellular level, ET is realized over nanometer length scales by assemblies of proteins that host redox-active cofactors, typically hemes^3,4^ or Fe–S clusters.^5,6^ These cofactors are meticulously arranged into chains within the protein matrix, separated by distances of <2 nm, where they act as stepping stones for the electrons to traverse the insulating protein. This electron “hopping” process is generally well described by the Marcus theory of nonadiabatic electron transfer.^7^ The respiratory complexes of the mitochondrial membrane^5,8^ as well as the multiheme cytochrome complexes of rock-respiring bacteria^3,9^ are key examples of this natural design principle.

Over the past two decades, it has however become clear that the spatial reach of biological electron transport may far exceed the nanometer length scale.^4,9,10^ The most striking example of such extremely long-range biological charge transport are cable bacteria. These multicellular bacteria are found in freshwater and marine sediments and capable of transferring electrons over centimeter scales.^11–14^ To this end, the cable bacteria embed a network of thin fibers (∼50 nm diameter) within their cell envelope.^15^ These fibers are arranged in a highly regular, equidistant and parallel pattern, and essentially act as a power line network that connects all cells within the centimeter-long bacterial filaments.

Intriguingly, these fibers from cable bacteria have been shown to exhibit electronic properties that are unprecedented in biology. Most prominently, electrical characterization revealed that the fibers display an extremely high conductivity (5–500 S cm^–1^),^16–19^ which substantially exceeds that of other known conductive biomaterials, like polymerized multiheme cytochromes (<50 × 10^–3^ S cm^–1^).^4,20,21^ As such, the fibers produced by cable bacteria may provide a promising design principle for novel materials^22^ within the emerging field of protein bioelectronics.^23–26^ Yet, the fundamental question of what biological electron transport mechanism is capable of sustaining such a high conductivity, remains presently unresolved.

Detailed electrochemical characterization has shown that the fiber conductance in cable bacteria exhibits conspicuous properties, including the absence of redox activity and the lack of a gating response.^18^ Likewise, the conductance shows a markedly weak thermal activation, with an activation energy of ∼40–50 meV at room temperature, which is 5 times lower than in multiheme cytochrome complexes.^27^ When the conduction is interpreted in terms of the conventional hopping picture, then very large effective transfer distances must be assumed (>10 nm) for the ET to remain within the classical nonadiabatic Marcus regime.^19^ This transfer distance is an order of magnitude larger than typical nearest neighbor distances in conductive proteins and other organic molecular materials, and would suggest that charge carriers are (partially) delocalized.^19^ As such, it has been argued that the charge transport in the fibers may be reminiscent of that in high mobility molecular organic semiconductors,^18,19^ where charge transport can occur via a transient delocalization mechanism with partially delocalized charge carriers.^28–39^ In single crystals of rubrene, holes can delocalize over multiple (>10) molecules at room temperature, which induces charge transfer over distances of 10 nm within the lattice.^35,38^ This length scale is hence similar to the one inferred for the transport in the conductive fibers of cable bacteria.

To attain a better mechanistic understanding of the charge transport in fibers of cable bacteria, one requires atomistic molecular models that serve as a structural basis for computational simulations.^40^ Very recently, considerable progress was made on this topic, which we will take advantage of here. Based on high-resolution microscopy and spectroscopy, it was shown that each fiber centrally embeds an entangled bundle of 10 nanoribbons.^41^ Each nanoribbon comprises a long, thin wire (1.4 nm diameter; >100 nm length), which is composed of nickel bis­(1,2-dithiolene) (NiBiD) oligomers as the molecular building block. In the model proposed, these planar molecules are axially aligned and slip-stacked into a multilayer, one-dimensional metal organic framework (MOF) that is a few molecular layers thick, thus forming the nanoribbon.^41^ The nanoribbons bear a strong resemblance to synthetic Ni-based coordination polymers, which exhibit comparably high electrical conductivities.^42^

These proposed nanoribbons provide a very new type of supramolecular MOF structure. Accordingly, it is adamant to properly characterize the electronic properties. To this end, we performed Density Functional Theory (DFT) calculations of prospective nanoribbons as to verify whether such structures can truly support the efficient conductance that has been measured in current–voltage measurements. Our aim was to obtain further insight into the molecular structure of the nanoribbons, as currently, certain aspects are not precisely known, such as the number of Ni centers in the NiBiD oligomers as well as the packing structure of these NiBiD units. Accordingly, we evaluated possible atomistic configurations and potential packing structures of nanoribbons, and ranked their stability by calculation of their cohesive energy. We then calculated the relevant electron transfer parameters governing charge transport in these generated structures, i.e., reorganization energy (λ) and electronic coupling (*H*_ab_). Both electron holes and excess electron transfer processes were considered because the dominant charge-carrier type has not yet been firmly established. These parameters provide a insight into the capability for efficient charge transport. More detailed first-principles-based calculations of charge mobility and conductivity are beyond the scope of this letter and are left for future work.

The nanoribbon structures evaluated were selected in accordance with the structural model as proposed in ref 41. The basic building block is the NiBiD unit shown in Figure 1a, which is a nickel ethylene tetrathiolate (Ni_3_(ett)_4_H_2_) compound containing three Ni centers terminated by two H atoms on opposite ends of the molecule. The electronic spin ground state of this molecule is a closed-shell singlet. While Raman spectroscopy indicates that multiple Ni centers are present,^41^ the actual value of *n* (the number of Ni centers) in the NiBiD oligomer remains uncertain. We selected *n* = 3 as a relevant, representative example, so that the molecule displays extended conjugation but is small enough to keep the simulation effort tractable. Likewise, the end-capping of the NiBiD oligomer is currently not resolved;^41^ two terminal H atoms are intended to represent potential sites for disulfide linkages to the protein environment. Comparison of simple -H capping versus -S-CH_3_ capping (which emulates cysteine capping as in ref.^41^) indicated that the electronic structure is largely insensitive to the identity of the terminating group (see ).

**Figure 1:**
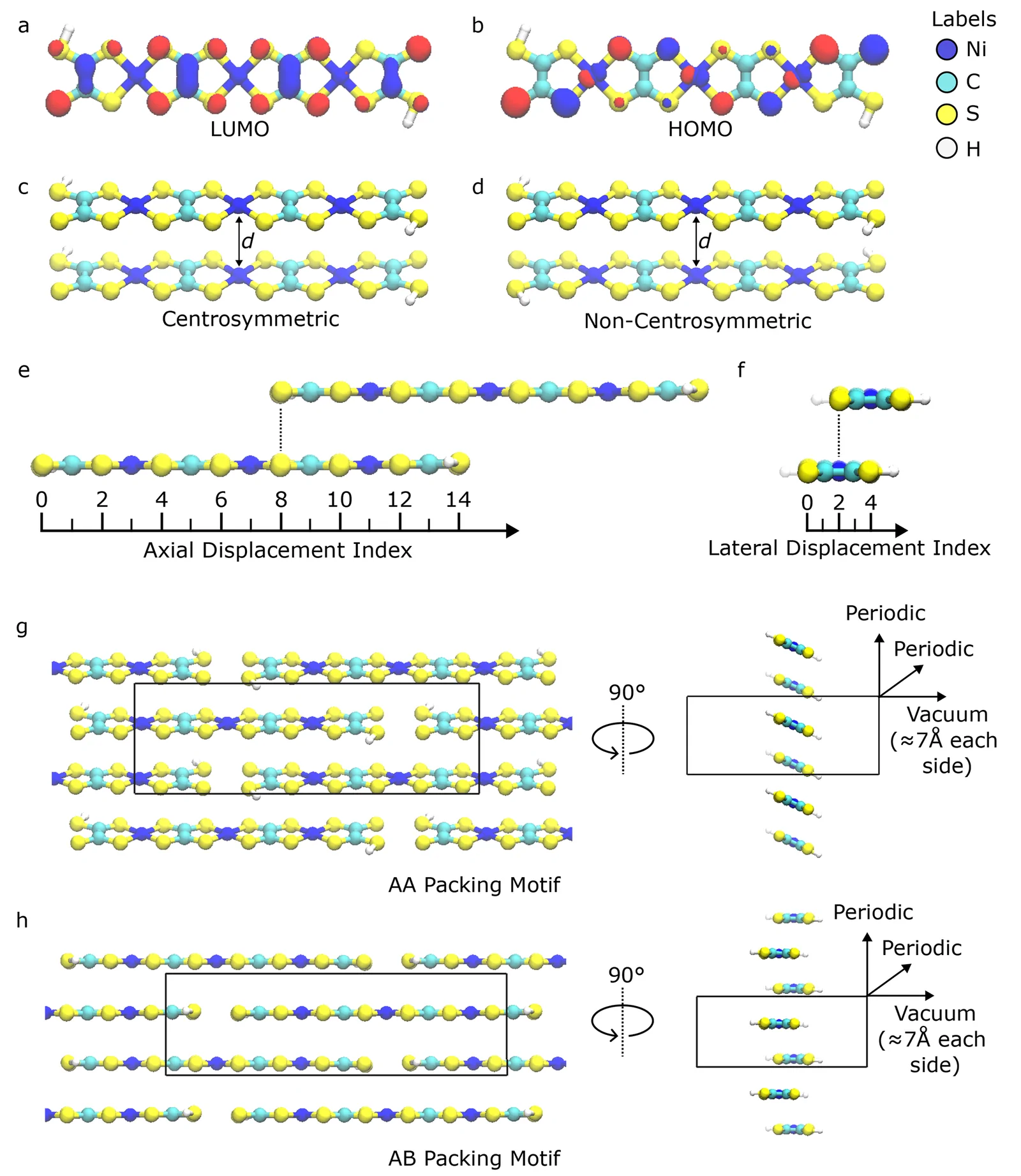
Frontier orbitals of the NiBiD monomer and structural parameters defining dimer and nanoribbon packing structures. Panels a and b show the LUMO and HOMO of the monomer, respectively, plotted at an isosurface value of ± 0.03, with red and blue denoting opposite orbital phases. Panels c and d define centrosymmetric and noncentrosymmetric configurations, respectively. Centrosymmetry refers to the presence or absence of inversion symmetry in a dimer pair, best indicated by the relative orientation of terminal H atoms. The interlayer stacking distance, *d*, is also indicated. Indexing schemes for axial and lateral displacements are shown in panels e and f, respectively. Each index corresponds to translating one monomer along the axial or lateral axis such that its terminal S atom aligns above the indexed atom, *N*, of the other monomer. These configurations are denoted Ax*N* and Lat*N*, as illustrated for Ax8 in (e) and Lat2 in (f). AA (g) and AB (h) packing motifs are shown from two viewing orientations, with the primitive unit cells indicating the periodic and vacuum directions. In AA packing, each layer is laterally shifted in the same direction relative to the layer below. In AB packing, the direction of this lateral shift alternates between layers.

Electronic structure calculations were carried out, unless stated otherwise, with a global hybrid functional based on PBE^43^ with 20% GGA exchange replaced by Hartree–Fock exchange (HFX), including the D3 dispersion correction^44^ and Becke-Johnson damping,^45^ denoted PBE20-D3­(BJ). The fraction of exact exchange was chosen such that Koopmans’ (Janak’s) theorem^46,47^ for the ionization potential and electron affinity of Ni_3_(ett)_4_H_2_ was obeyed (see ). However, calculations with the standard PBE0 (25% HFX)^48^ functional gave similar results, suggesting that simulation results are robust against the choice of HFX fraction.

The optimization of nanoribbon packing structures required a large number of total energy and force calculations, which are computationally expensive with hybrid functionals. Thus, nanoribbon packing structures and cohesive energies were calculated using PBE-D3­(BJ). This functional gave good performance on a benchmark set of small aliphatic and aromatic dimers with a mean absolute deviation ratio (MADR) compared to CCSD­(T) of 9% (similarly, PBE0-D3­(BJ) MADR = 11%).^49^ All calculations were carried out with the CP2K program package.^50^ In the following we present calculations on monomer and dimer structures of Ni_3_(ett)_4_H_2_ before presenting results for extended nanoribbon structures involving multiple stacked units.

The LUMO and HOMO of Ni_3_(ett)_4_H_2_ are shown in Figure 1a,b, respectively. Both orbitals are primarily located on the ett ligands with very little or no contribution from the Ni d states. Notably, the LUMO is approximately C_2_-symmetric about the long and short molecular axes and the axis perpendicular to the molecular plane. The orbitals are qualitatively very similar to those reported in ref 41 for the same oligomer with the B3LYP functional, and resemble those of related Ni-based dithiolene complexes as reported in refs 51 and 52. The reorganization energy for charge transfer between two Ni_3_(ett)_4_H_2_ molecules (at infinite separation) is 158 meV for electrons and 109 meV for holes, with the largest structural changes being the contraction of the central C–C bond and Ni–S bond of the dithiolene rings, respectively for electrons and holes (all <0.03 Å). A smaller excess electron reorganization energy of 40 meV has been reported for Ni_3_(ett)_4_H_2_ with the GGA functional BP86.^40^ However, it is well-known that GGA functionals tend to underestimate reorganization energy due to their deficiency in underestimating the energy for bond distortion.^34,53^ Replacing the terminal H atoms by S-CH_3_, the reorganization energy changes only very little, 162 meV for electrons and 119 meV for holes. Overall, the predicted reorganization energies are typical of organic pi-conjugated molecules.^34^

Electronic couplings in Ni_3_(ett)_4_H_2_ dimers for electrons and holes were calculated using the projection operator based diabatization (POD) method (available in the CP2K package; see refs 54 and 55 for theoretical details). When dimers are positioned in perfect cofacial alignment, the electronic coupling decays exponentially with separation distance with an exponential distance decay constant of 2.9 Å^–1^ (noncentrosymmetric alignment) and 3.1 Å^–1^ (centrosymmetric alignment) for holes and 2.6 Å^–1^ in both alignments for electrons, Figure 2a,b. These values are again typical for organic pi-conjugated molecules.^55–57^ Here, (non)­centrosymmetric refers to absence or presence of inversion symmetry in the dimer pair due to the relative orientation of the H atoms on each monomer, as defined in Figure 1c,d. Electronic coupling for electrons is the same for both H atom orientations due to the approximate *C*_*2*_ symmetry of the LUMO.

**Figure 2:**
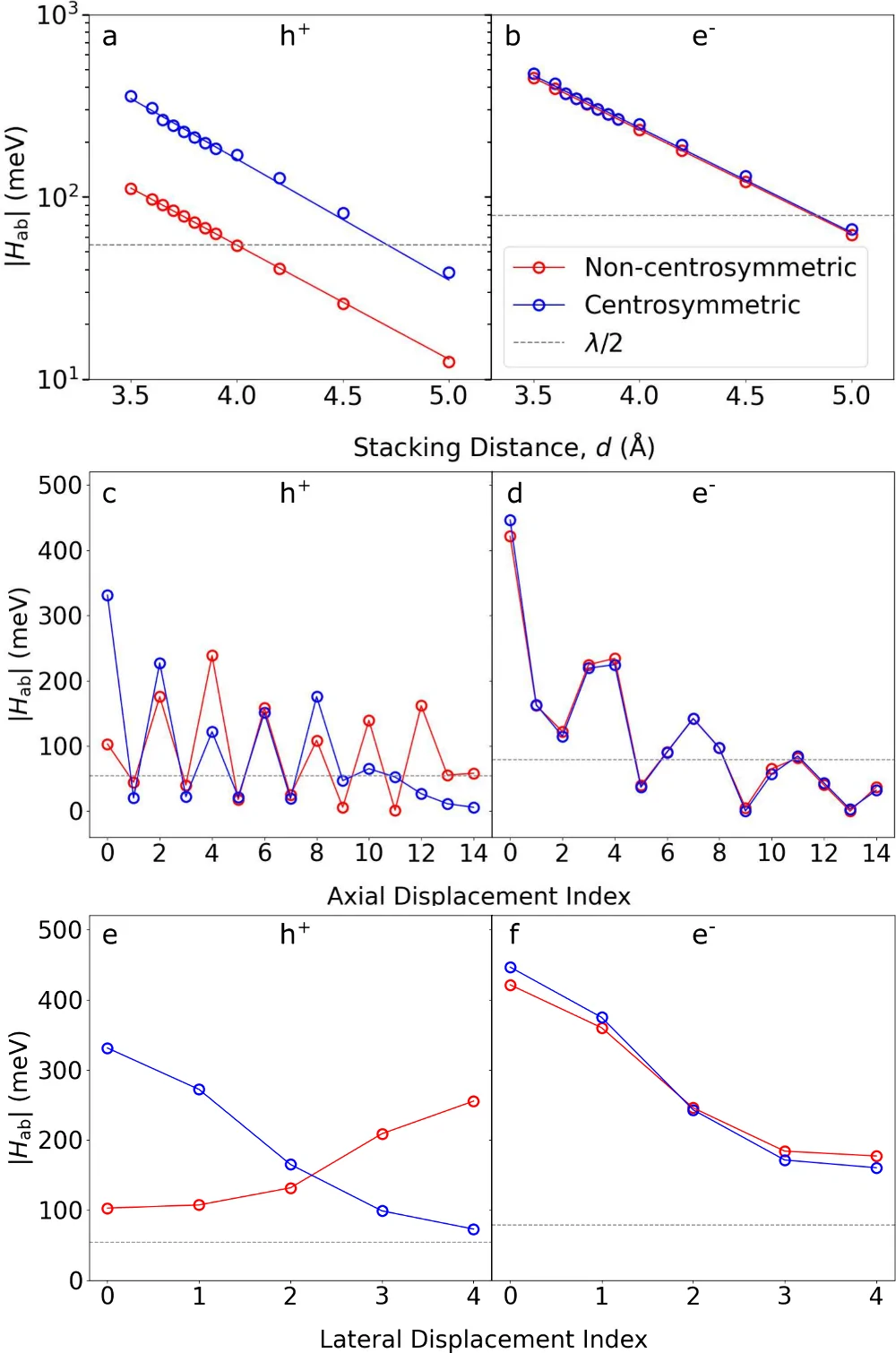
Electronic coupling of NiBiD dimers in vacuum in centrosymmetric and noncentrosymmetric configurations. Electronic coupling for hole (HOMO–HOMO, (a) and excess electron (LUMO–LUMO, (b) for the fully cofacial dimer pair (Ax0Lat0) is shown as a function of interlayer stacking distance, *d*. The distance dependence is fitted to |*H*_ab_| = *A*e^–*βd*/2^, giving decay constants of 2.9 and 3.1 Å^–1^ for hole coupling in the noncentrosymmetric and centrosymmetric configurations, respectively, and 2.6 Å^–1^ for excess-electron coupling in both configurations. Hole (c) and excess electron (d) coupling are plotted against axial displacement index at a fixed interlayer stacking distance (3.55 Å) and lateral displacement (Lat0). Similarly, hole (e) and excess electron (f) coupling are plotted against lateral displacement index at fixed interlayer stacking distance (3.55 Å) and axial displacement (Ax0; see Figure 1e,f for definition of the displacement index). The threshold λ/2, where λ is the reorganization energy for hole or excess electron transport, is also shown; coupling values above this threshold are typically associated with efficient charge transport via the transient delocalization mechanism.

Next we investigated the effect of displacing one monomer with respect to the other along the long and short molecular axes, respectively referred to as “axial” and “lateral” displacement. The axially and laterally displaced dimer configurations are labeled Ax*N* and Lat*N*, respectively. The displacement index *N* indicates the magnitude of the shift versus the reference cofacial alignment: *N* = 0, ..., 14 in the axial direction (Figure 1e) and *N* = 0, ..., 4 in the lateral direction (Figure 1f). The electronic coupling of the axially displaced configuration exhibits a distinctive zigzag pattern (Figure 2c,d) which is particularly pronounced for holes where maxima occur when S atoms of the two NiBiD units are on top of one another (Figure 2c). In contrast, for laterally displaced monomers, the electronic coupling steadily decreases or increases with lateral index (Figure 2e,f). All trends in electronic coupling can be readily explained in terms of constructive and destructive frontier orbital overlaps, which sensitively depend on the axial and lateral displacements; this is well established for other pi-conjugated dimers.^58^ Crucially, the dimer calculations show that in certain dimer configurations high electronic couplings are obtained, which exceed the critical threshold for charge delocalization, *H*_ab_ > λ/2 (indicated by a dashed line in Figure 2a−f). These high electronic couplings persist even at relatively large axial and lateral displacements. Future microscopy and spectroscopic investigations should examine whether Ni_3_(ett)_4_H_2_ nanoribbons preferentially pack into such electronically strongly coupled molecular configurations. Since the effective packing structure of nanoribbons remains unknown, we may take some clues from comparable Ni-bis­(dithiolene) materials for which the packing structure has been experimentally determined.^42^ These materials tend to form two packing motifs, denoted AA and AB. These motifs correspond to the direction of translation along the nonperiodic lateral axis, which can either occur in the same direction (AA; Figure 1g) or alternate between adjacent layers (AB; Figure 1h).

We constructed unit cells containing two Ni_3_(ett)_4_H_2_ molecules in their centrosymmetric and noncentrosymmetric orientation. The molecular structure was determined from geometry optimization of the single molecule in vacuum using the PBE-D3­(BJ) functional. In a first coarse scan of possible AA and AB packing structures, we generated axially and laterally displaced dimers at a fixed stacking distance corresponding to the potential energy minimum for the dimer in vacuum (≈3.55 Å; see ). The unit cell vectors were chosen such that the axial, lateral and stacking distances between monomers within and across the unit cell boundaries were equal. The unit cell was then periodically replicated in the axial and in the stacking direction, but not in the lateral direction. According to the spectroscopic data, nanoribbons consist of 2–5 molecular layers in the stacking direction,^41^ and hence, they are finite along the stacking direction. Still, we also applied periodic boundary conditions along the stacking direction to avoid artificial surface effects that would otherwise be introduced. Given that we chose displacements between all neighboring monomers to be equal, axial displacements smaller than axial index 8 are inherently energetically unfavorable due to steric overlap between molecules of adjacent cells (see ). To quantify the relative stability of each packing structure, we calculated the cohesive energy, *E*_coh_ (defined positively), using the PBE-D3­(BJ) functional,

1 $$E_{\mathrm{coh}}=-\left(E_{\mathrm{tot}}-NE_{\mathrm{mono}}\right)$$

where *E*_mono_ is the energy of the monomer in vacuum, *E*_tot_ is the total energy of a unit cell and *N* is the number of monomers in the unit cell (*N* = 2 unless stated otherwise). The greater the cohesive energy, the more energetically stable and thus likely a given configuration is.

Figure 3 displays the cohesive energies for AA and AB packed structures as a function of the lateral displacement (Lat0–Lat4) for different axial displacements (Ax8–Ax12) at a constant stacking distance. The cohesive energy is governed by the balance between dispersion interactions and Pauli repulsion. In both the AA and AB packed structures, the configurations with the smallest axial displacements (Ax8 and Ax9), have the greatest dispersion interaction and cohesive energy for all lateral displacements investigated. Furthermore, a preference for lateral displacements Lat2 and Lat3 is observed, which can be attributed to reduced orbital overlap and, therefore, decreased Pauli repulsion, despite slightly weaker dispersion interactions. In addition, AB packing structures are generally more stable than their AA counterparts, owing to the shorter interlayer next-nearest-neighbor distance in AB, which provides additional dispersion stabilization (see ). Overall, this coarse scan identified the Ax8 and Ax9 packed structures as the most stable configurations across both AA and AB packing motifs in both centrosymmetric and noncentrosymmetric configurations.

**Figure 3:**
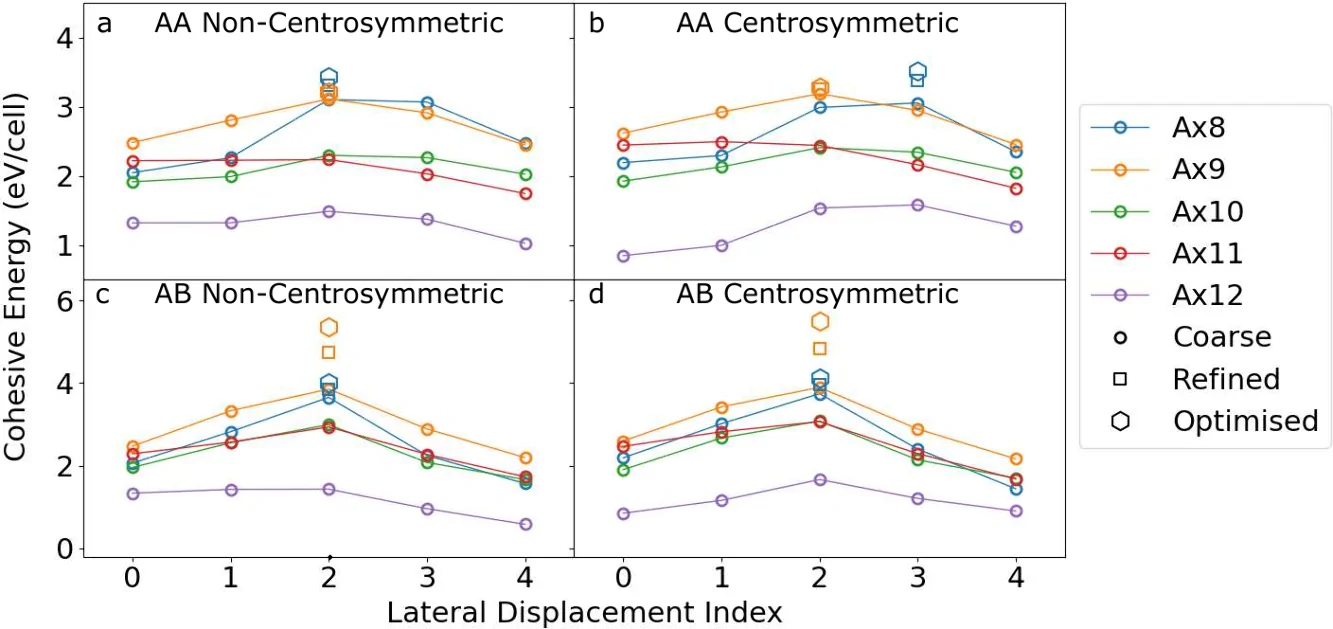
Packing stability for 2D NiBiD nanoribbon model structures. Cohesive energies per unit cell, calculated using PBE-D3­(BJ), are shown for 2D nanoribbons with different packing motifs (AA and AB) and dimer inversion symmetry configurations (centrosymmetric and noncentrosymmetric): (a) AA noncentrosymmetric, (b) AA centrosymmetric, (c) AB noncentrosymmetric, and (d) AB centrosymmetric. Coarse points (circles) correspond to structures generated across axial displacement indexes Ax8–Ax12, indicated by color, and lateral displacement indexes Lat0–Lat4, shown on the *x*-axis. ‘Refined’ points (squares) and ‘Optimized’ points (hexagons) correspond to structures generated by a refinement procedure and subsequent geometry optimization of the refined structure, respectively. These points are plotted using the same indexing scheme (color and *x*-axis) as the coarse structure from which refinement was initiated. See Figure 1e,f for definition of displacement index.

In a subsequent step, we refined the AA and AB packings and the corresponding unit cell dimensions starting from configurations with the greatest cohesive energy in the Ax8 and Ax9 series (Lat2 or Lat3) by varying the axial and lateral displacements and the stacking distance in increments of 0.01 Å. Despite refined structures no longer aligning with the initial axial and lateral displacement indexes, we continue to refer to them with the indexes of the structure from which each refinement was initialized (e.g., refined Ax8). AB motifs remained more stable than AA motifs upon refinement. Yet, the magnitude of the increase in cohesive energy varied across configurations, with modest stabilization upon refinement for AA Ax8, AA Ax9, and AB Ax8 initial structures, but a marked increase in cohesive energy for AB Ax9 initial structure (Figure 3, data points indicated by squares). Analysis of the refined AB Ax9 configuration indicated that the large increase in cohesive energy originated from alignment of Ni and S atoms between layers, accompanied by a significant reduction in interlayer separation from ≈3.5 to 3.0 Å, enabling the formation of a weak axial Ni–S coordination bond. Similar Ni–S interactions have been highlighted in other Ni-based dithiolene complexes.^42^ Such interlayer contacts are geometrically not possible in Ax8 configurations. Interestingly, they would be possible in AA Ax9, but are energetically not favorable in this configuration.

Final “optimized” structures were obtained by geometry optimization of the refined structures under fixed unit cell parameters. AB Ax9 underwent marked structural relaxations along with large energetic stabilization (Figure 3c,d, hexagons in orange), whereas the other structures exhibited minimal relaxations and energetic stabilization (AA Ax8, AB Ax8 blue hexagons Figure 3a–d; AA Ax9 orange hexagons Figure 3a,b). In AB Ax9, Ni and S atoms involved in close interlayer contacts distorted out of their NiS_4_ square planes upon optimization, giving rise to shortened interlayer Ni–S distances (≈2.5 Å) with the lower plane, resulting in the formation of a 5-fold coordinated Ni center, and longer interlayer Ni–S distances with the upper plane (≈3.5 Å) (see Figure 4c). Each monomer can form 4 such interactions (2 at each end) with neighboring monomers in the layers above or below. Conspicuously, in the optimized AB Ax9 stacking, the terminal ends of the symmetric NiBiD molecules behaved differently. The right-hand side formed interlayer coordination interactions with both layers above and below, whereas the left-hand side formed interactions only with the layer below. This resulted in symmetry breaking between monomers and the emergence of nonequivalent sites. Across all packing structures analyzed, AB Ax9 packing emerged as the most stable configuration, owing to its unique ability to form interlayer Ni–S coordination through structural reorganization (Figure 4a,c), with AB Ax8 being the second most stable structure (Figure 4b,d).

**Figure 4:**
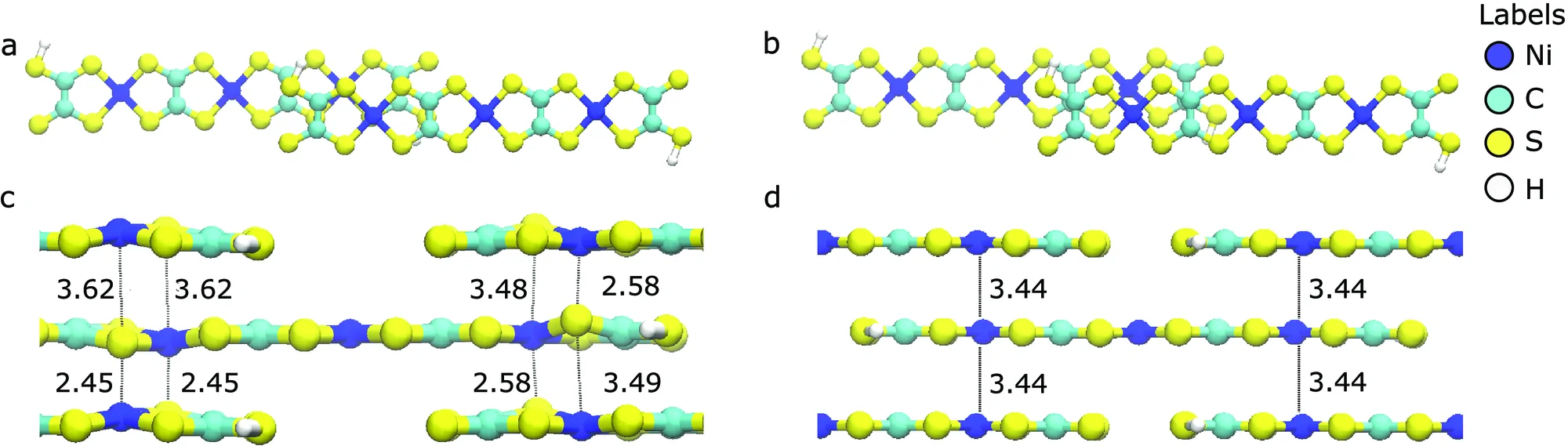
Structural comparison of optimized centrosymmetric AB Ax9 and AB Ax8 nanoribbon structures. Dimer pairs extracted from optimized 2D nanoribbon sheets are shown from above for centrosymmetric AB Ax9 (a) and AB Ax8 (b). The optimized AB Ax9 structure shows clear interlayer alignment of Ni and S atoms; this alignment is not observed in the optimized AB Ax8 structure. Snapshots of the corresponding 2D nanoribbon are shown in (c) and (d) for optimized AB Ax9 and AB Ax8, respectively. In (c), the Ni and S atoms distort out of their NiS_4_ square planes, producing shorter and longer interlayer Ni–S distances whereas the shorter distances correspond to interlayer Ni–S bonds. This reduction in interlayer distance is not observed in (d), where the individual Ni_3_(ett)_4_H_2_ units remain undistorted and planar.

While the cohesive energy determines the packing structure of the NiBiD molecule, the electronic coupling governs its charge transport capability. Thus, it is of interest to evaluate the electronic coupling for the different packing structures generated. In the standard approach for calculating electronic couplings in the condensed phase, one extracts molecular dimers from the bulk and carries out coupling calculations on them in vacuum. We found that this approach was not applicable for Ni_3_(ett)_4_H_2_ because the diabatic frontier orbitals of the dimer exhibited strong polarization with respect to the frontier orbitals in the single monomer in vacuum (). This polarization does not happen in the periodic environment of a nanoribbon, where the diabatic frontier orbitals resemble closely the frontier orbital of the single monomer in vacuum (). Thus, we computed electronic couplings between neighboring molecules directly in the condensed phase using periodic boundary conditions exploiting the unique capabilities of the POD method (see ).

For both hole and excess electron coupling, Ax8 configurations (Figure 5, data in blue) exhibited significantly larger couplings than Ax9 configurations (Figure 5, data in orange), consistent with the frontier orbital overlap arguments used above to explain the trends for dimers in vacuum (Figure 2). For most configurations, refinement and geometry optimization had little effect on the coupling values as the structures did not deviate significantly from their initial geometries (Figure 5, data indicated by squares and hexagons, respectively). As with cohesive energy, AB Ax9 was the outlier. A marked increase in electronic couplings was observed upon refinement (Figure 5b,d, squares in orange) which was driven by the significantly reduced stacking distance (axial Ni–S distances ≈3.0 Å). In the optimized structure, the formation of short and long axial Ni–S distances (≈2.5 and ≈3.5 Å, Figure 4c), resulted in 4 nonequivalent couplings, one with a very large value corresponding to the dimer pair with two short axial Ni–S bonds and three with smaller values (Figure 5b,d, hexagons in orange). While the precise pattern of short and long axial Ni–S distances may depend on the thickness of the nanoribbon, they will always result in the emergence of nonequivalent electronic couplings.

**Figure 5:**
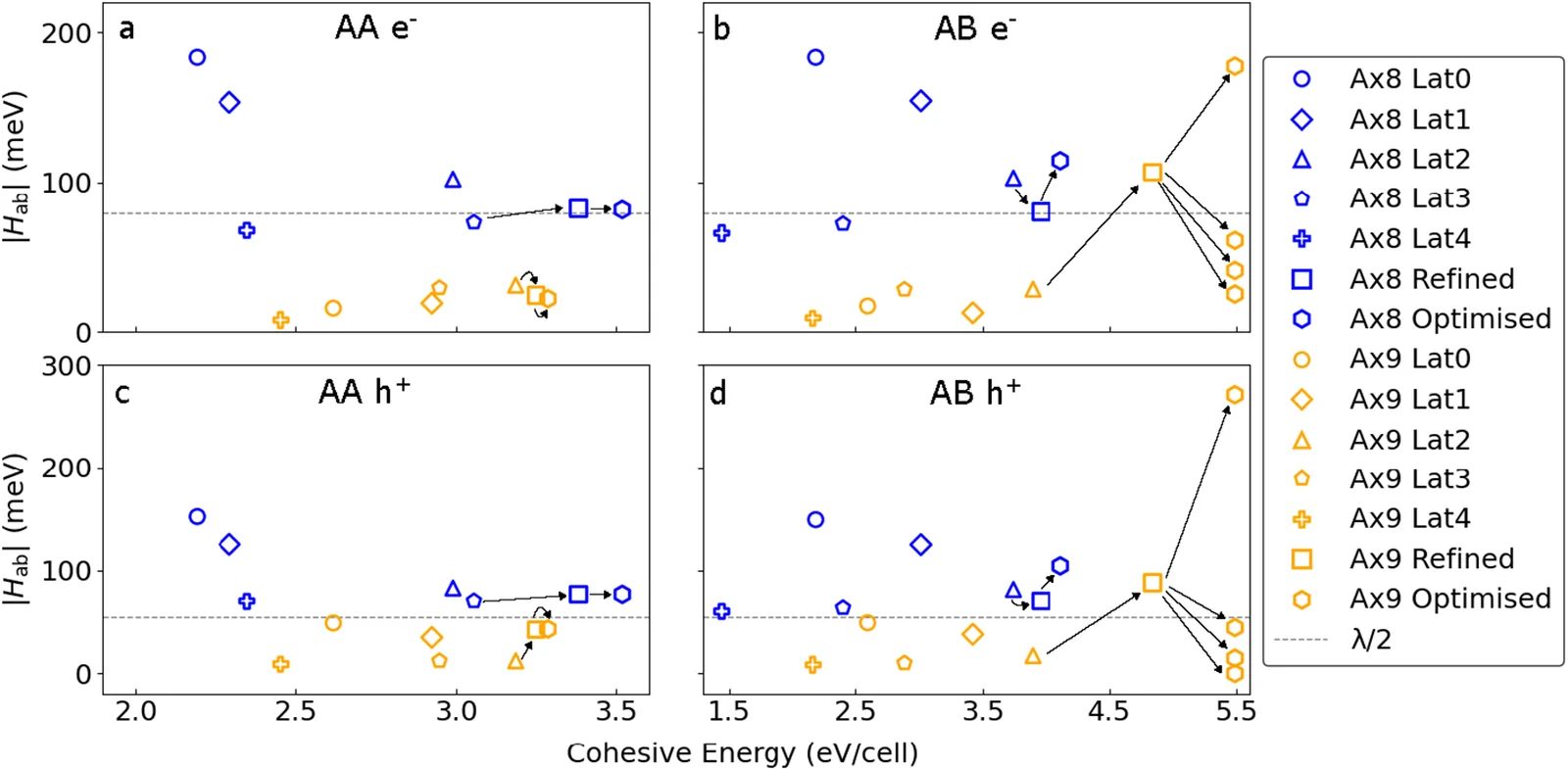
Electronic coupling versus cohesive energy for centrosymmetric nanoribbon model structures. Electronic coupling (*y*-axis) and cohesive energy (*x*-axis) are plotted for centrosymmetric packing structures, with color indicating axial displacement index and marker shape indicating lateral displacement index. Refined and optimized structures are shown as squares and hexagons, respectively. AA and AB packing motifs are shown for excess electron coupling in panels a and b, respectively, and for hole coupling in panels c and d, respectively. Dashed gray lines indicate the λ/2 threshold, as in Figure 2. Black arrows indicate the progression from ‘Coarse’ to ‘Refined’ structures (triangle to square) and from ‘Refined’ to ‘Optimized’ structures (square to hexagon). Analogous data for noncentrosymmetric configurations are shown in .

Efficient charge transport in the transient delocalization regime occurs when the electronic coupling exceeds λ/2 (Figure 5, dashed lines). Using this criterion, a clear contrast emerges between AB Ax8 and AB Ax9 configurations. In the AB Ax8 configuration each molecule is connected with its neighbors by couplings that exceed this threshold, for both excess electron and hole. In this way, continuous strong coupling paths exist that support efficient delocalized transport. By contrast, in the AB Ax9 configuration only one molecular pair is strongly coupled with a value exceeding λ/2 whereas others are weakly connected with values below λ/2. Thus, this structure does not form a continuous path of strong electronic couplings exceeding λ/2, yet delocalized transport may still occur depending on the extent of eigenstate delocalization and thermal electronic disorder. Investigation of such effects was however beyond the scope of this work and is left for future investigations.

Several previous experimental and computational studies of NiBiD-related complexes have provided detailed insight into molecular electronic structure and packing motifs.^42,51,52,59,60^ However, simultaneous consideration of packing stability and intermolecular electronic coupling has not been carried out, to our best knowledge. Here, a clear trade-off between structural stability and charge transport is revealed within the NiBiD nanoribbons investigated. While the AB Ax9 configuration is the most stable structure due to its ability to form interlayer Ni–S coordination interactions, this same structural reorganization leads to asymmetric environments and nonequivalent couplings that may limit charge transport. In contrast, Ax8 configurations are less energetically stabilized, but maintain configurations that preserve a good orbital overlap between neighboring molecules. This gives rise to a continuous network of high electronic couplings exceeding λ/2 and enabling charge delocalization. Yet, it is important to note that in our idealized simulations, we considered only nanoribbons consisting of Ni_3_(ett)_4_H_2_ oligomers in isolation, and hence we did not account for possible interactions with the surrounding environment, such as a protein scaffold, which is proposed to encapsulate the nanoribbons in the periplasm of cable bacteria.^61^ These environmental effects could tip the energetic balance between the different packing motifs and might stabilize configurations with favorable continuous electronic coupling networks that are not the most stable in isolation, such as the AB Ax8 structures investigated here.

In conclusion, we have evaluated a possible set of atomistic model structures for cable bacterial nanoribbons made of NiBiD oligomers. Some of these configurations support efficient delocalized electron transport beyond nearest-neighbor hopping, which could help explain the high conductivity and other unusual electrical properties (no redox activity, weak gating response, weak thermal activation) that are reported for these intriguing biological structures.^16,18,19,62^ Future work should concentrate on the further experimental elucidation of the atomistic structure of the nanoribbons, which could provide a validation of the exploratory simulation results presented here. The current work has laid the foundation for state-of-the-art nonadiabatic dynamics simulations^34–38^ of NiBiD oligomer-based nanoribbons, which will quantitatively elucidate the intrinsic charge mobilities and transport mechanism.

## Supplementary Material





## Notes

NiBiD coordinates (ZIP)
Full computational details on the preparation of nanoribbons, frontier orbitals, optimal Hartree–Fock exchange fraction, bond lengths and HOMO–LUMO gaps, interlayer stacking distance, unit cell construction and constraints, AA vs AB cohesive energy, electronic coupling (PDF)
