# Supplementary material for: Model Structures and Electron Transfer Properties of Conductive Nickel-Organic Nanoribbons in Cable Bacteria
Source: J Phys Chem Lett. 2026 Aug 31;17(36):10340–8. doi: 10.1021/acs.jpclett.6c01926 (PMC13573619; doi:10.1021/acs.jpclett.6c01926)
Supplement: Supplementary file 1 [file jz6c01926_si_002.pdf]

Supporting Information: Model structures and electron transfer properties of conductive nickel-organic nanoribbons in cable bacteria

Oliver Russell,[†,‡] Martijn A. Zwijnenburg,[¶,‡] Filip J.R. Meysman,[§] and Jochen Blumberger*,[†,‡]

†*Department of Physics and Astronomy, University College London, London, WC1E 6BT, United Kingdom*

‡*Thomas Young Centre, London, WC1E 6BT, United Kingdom*

¶*Department of Chemistry, London, WC1H 0AJ, United Kingdom*

§*Department of Biology, University of Antwerp, Antwerp 2610, Belgium*

E-mail: j.blumberger@ucl.ac.uk

Monomer orbital comparison

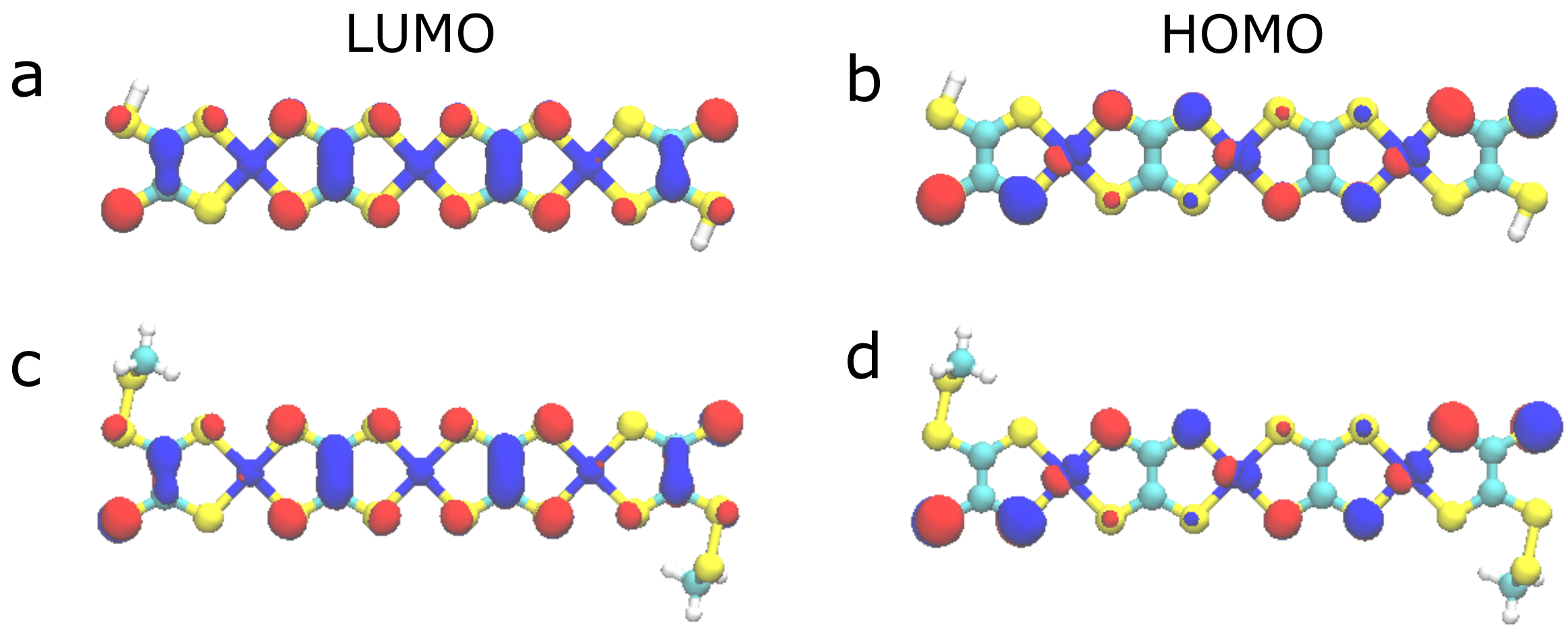

Figure S1: **Frontier orbitals of $(Ni_3(ett)_4H_2)R_2$ with -H and -SCH_3 terminating groups (R). a** and **b** show the LUMO and HOMO, respectively, for R = -H; **c** and **d** show the LUMO and HOMO, respectively, for R = -SCH_3. Red regions correspond to an isosurface value of -0.03; blue regions correspond to +0.03. The LUMO and HOMO distributions are qualitatively insensitive to the identity of the terminating group.

Optimization of Hartree-Fock exchange fraction and assessment of DFT+U

As outlined in Ref.,[1] the fraction of Hartree-Fock exchange (HFX) in a hybrid functional can be tuned by enforcing Koopmans' (Janak's) theorem:[2,3]

$$I = -\varepsilon^{0}_{\mathrm{HOMO}} \tag{S1}$$

$$A = -\varepsilon^{-}_{\mathrm{HOMO}} \tag{S2}$$

where I and A are the vertical ionization energy and electron affinity, respectively. Here, $\varepsilon^{0}_{\mathrm{HOMO}}$ is the HOMO energy of the neutral monomer and $\varepsilon^{-}_{\mathrm{HOMO}}$ is the majority-spin HOMO energy of the anion. The corresponding quantities are given by:

$$I(\alpha) = E^{+}(\alpha) - E^{0}(\alpha) \tag{S3}$$

$$A(\alpha) = E^{0}(\alpha) - E^{-}(\alpha) \tag{S4}$$

where $E^{+}(\alpha)$, $E^{0}(\alpha)$ and $E^{-}(\alpha)$ are the total energies of the cation, neutral and anion, respectively, with α referring to the percentage of HFX and each evaluated in the neutral geometry. The optimal fraction of HFX was determined by minimizing the square difference,$J(\alpha)$, between orbital energies and the corresponding ionization energies and electron affinities, thereby enforcing Koopmans' (Janak's) condition.

$$DI(\alpha) = E^{+}(\alpha) - E^{0}(\alpha) + \varepsilon^{0}_{\mathrm{HOMO}}(\alpha) \tag{S5}$$

$$DA(\alpha) = E^{0}(\alpha) - E^{-}(\alpha) + \varepsilon^{-}_{\mathrm{HOMO}}(\alpha) \tag{S6}$$

$$J(\alpha) = DI^{2}(\alpha) + DA^{2}(\alpha) \tag{S7}$$

As shown in Figure S2, the optimal fraction of HFX for a single NiBiD molecule in vacuum was found to be 20%.

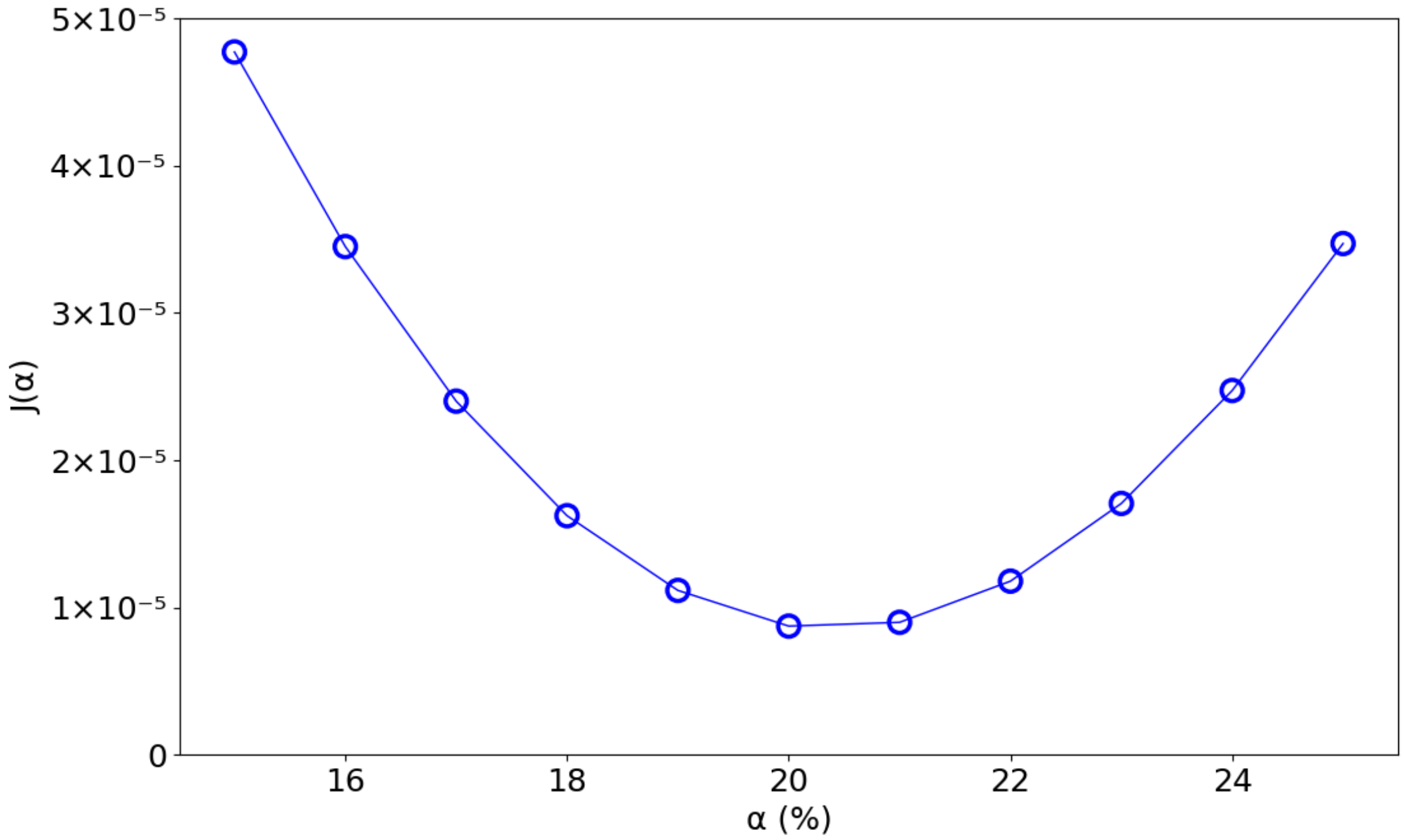

Figure S2: **Optimal Hartree-Fock exchange fraction.** Using Koopmans' theorem, the square deviation between ionization energy (electron affinity) and orbital energy, $J(\alpha)$, is shown as a function of the HF exchange fraction, α. A minimum in $J(\alpha)$ is found at $\alpha = 20\%$.

Additionally, DFT+U[4] calculations were performed to assess whether the treatment of localized Ni 3d states significantly affects the geometry and electronic structure of the NiBiD molecule. Application of the Hubbard correction produced a systematic elongation of the Ni-S bonds, with the mean bond length increasing from 2.169 Å at the PBE level to 2.185 Å at $U_{\mathrm{eff}} = 3$ eV and 2.206 Å at $U_{\mathrm{eff}} = 6$ eV. In contrast, the mean Ni-S bond length obtained using PBE20 was 2.167 Å, indicating that inclusion of Hartree–Fock exchange has little effect on the optimized Ni–S geometry. The HOMO–LUMO gap also increased from 0.228 eV with PBE to 0.594 eV at $U_{\mathrm{eff}} = 3$ eV, before remaining approximately constant at 0.69 eV for $U_{\mathrm{eff}} \geq 4$ eV. These values remain substantially smaller than the PBE20 gap of 1.482 eV, demonstrating that DFT+U and Hartree-Fock exchange affect the electronic structure differently (Table S1).

At $U_{\mathrm{eff}} = 3$ eV, the HOMO and LUMO retain the same qualitative spatial character as

those obtained using PBE20, remaining delocalized across the dithiolene framework rather than becoming localized on the Ni centers (Figure S3). Therefore, although DFT+U produces quantitative changes in the Ni-S bond lengths and HOMO-LUMO gap, a moderate Hubbard correction does not qualitatively alter the frontier electronic structure or the resulting interpretation of the NiBiD system.

Table S1: Mean Ni-S bond lengths and HOMO–LUMO gaps calculated using PBE, DFT+U and the optimized PBE20 hybrid functional. The effective Hubbard parameter, $U_{\text{eff}} = U - J$, was applied to the Ni 3d orbitals.

Method	Mean Ni-S bond length (Å)	HOMO–LUMO gap (eV)
PBE	2.169	0.228
$U_{\text{eff}} = 3$ eV	2.185	0.594
$U_{\text{eff}} = 4$ eV	2.192	0.690
$U_{\text{eff}} = 5$ eV	2.199	0.688
$U_{\text{eff}} = 6$ eV	2.206	0.685
PBE20	2.167	1.482

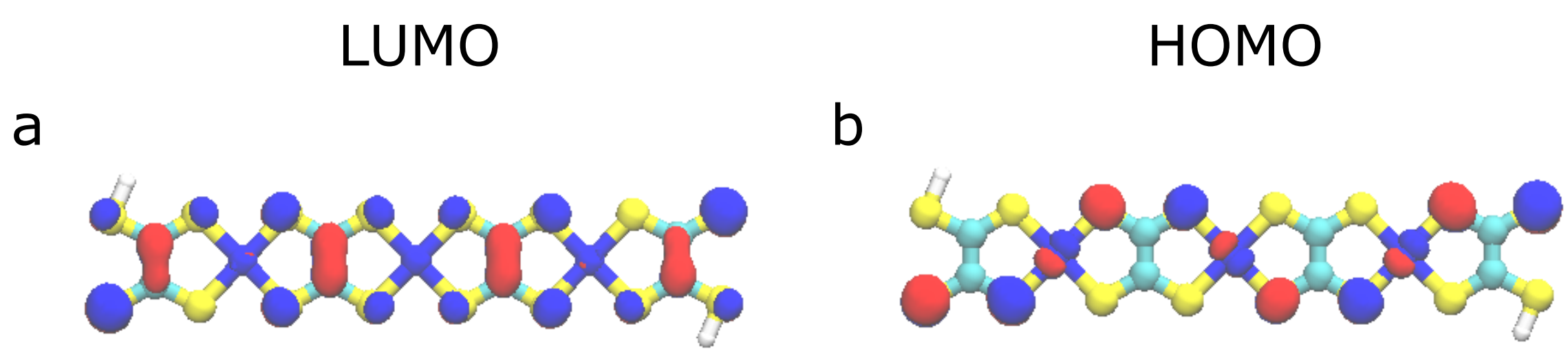

Figure S3: **Frontier orbitals of $(Ni_3(ett)_4H_2)H_2$ calculated using DFT+U with $U_{\text{eff}} = U - J = 3$ eV. a** and **b** show the LUMO and HOMO, respectively. Red regions correspond to an isosurface value of −0.03; blue regions correspond to +0.03.

Determination of interlayer separation distance

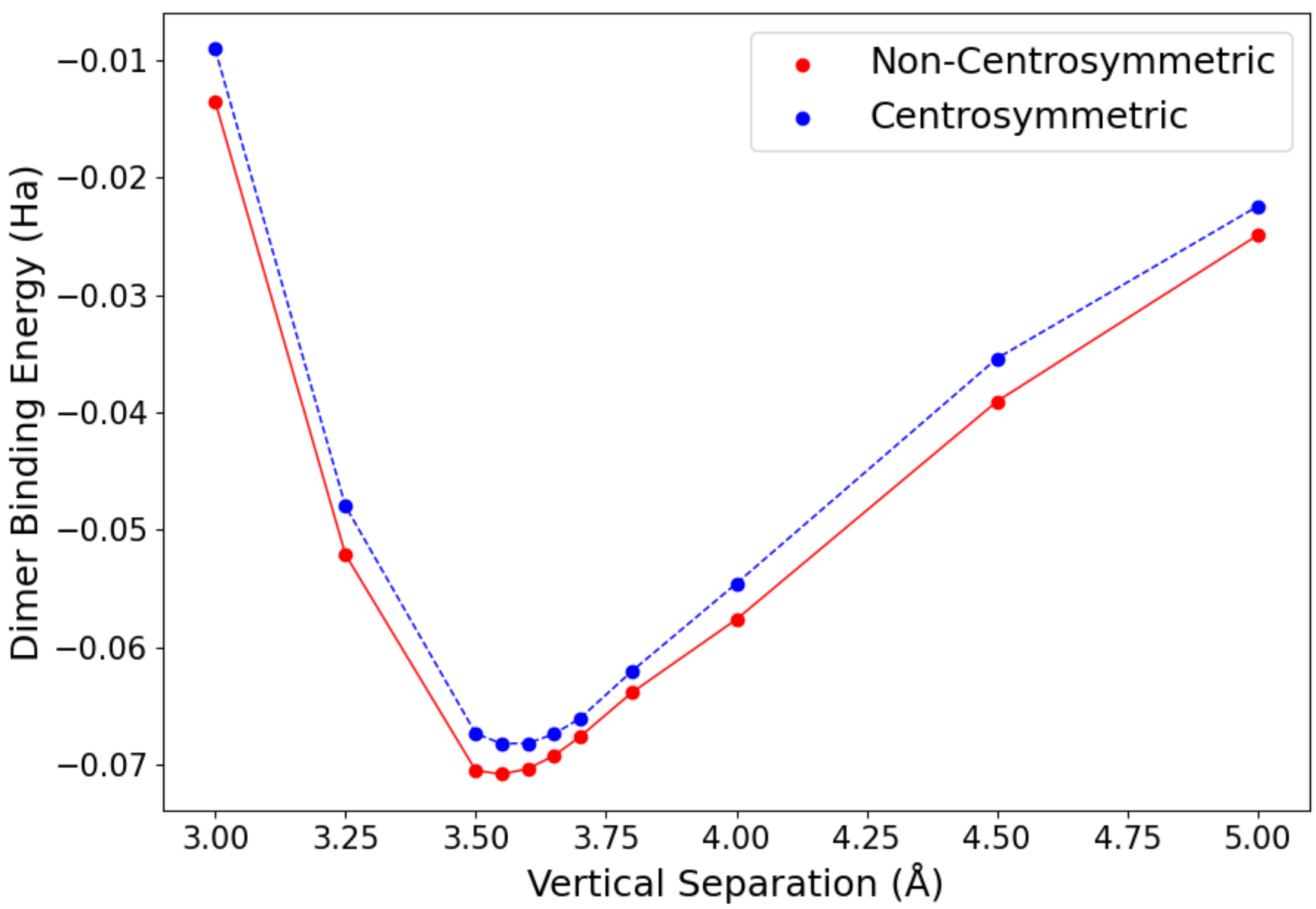

Figure S4: **Interlayer stacking distance.** The dimer binding energy for fully cofacial dimer pairs is shown as a function of interlayer stacking distance. For both centrosymmetric and non-centrosymmetric configurations, minima are found at ≈ 3.55 Å.

In order to compare axial and lateral displacement, it is necessary to first establish a constant interlayer separation distance. By taking the perfect co-facial dimer pair, we vary the interlayer separation distance (as in Figures 2a and 2b) and evaluate the dimer binding energy, E_{dim} (defined negatively).

$$E_{\text{dim}} = E_{\text{tot}} - 2E_{\text{mono}} \tag{S8}$$

This was computed using the PBE-D3(BJ) functional, consistent with cohesive energy calculations, and is shown in Figure S4. Both centrosymmetric and non-centrosymmetric

orientations present a minima at ≈ 3.55 Å, hence for all calculations, unless otherwise stated (refined and optimized), an interlayer separation of 3.55 Å was used.

Unit cell construction and constraints

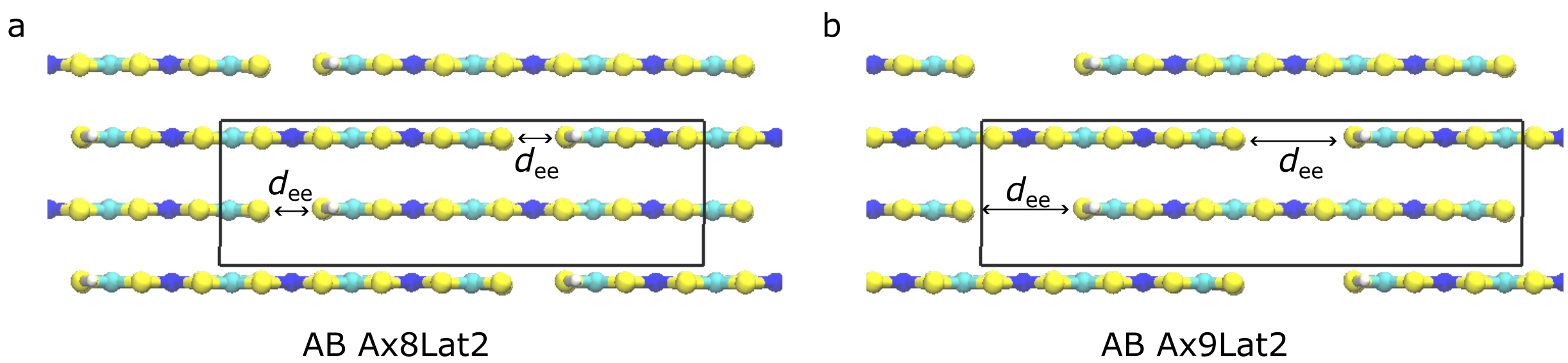

Figure S5: **Intralayer NiBiD unit distance.** Intralayer distances, d_{ee}, between the ends of NiBiD molecular units are shown for packing structures with axial translations to axial index 8 (**a**) and axial index 9 (**b**).

The intralayer NiBiD end-end distance, d_{ee}, is introduced in Fig.S5. The end-end distance is controlled by axial displacement, with larger axial displacements giving larger end-end distances (see Ax8 and Ax9 in Fig.S5). This follows from the imposed condition of equal displacement between neighboring monomers within and across the unit cell boundaries. The intralayer end-end distance, d_{ee} is given by:

$$d_{ee} = 2d_{ax} - L_{mono} \tag{S9}$$

where d_{ax} is the axial displacement and L_{mono} is the length of an individual NiBiD unit in the axial direction (21.036 Å). The resulting intralayer end-end distances are outlined for different axial displacement indexes in Table S2.

Table S2: Intralayer end-end distances in packing structures constructed with different axial displacements.

Axial Index	d_{ax} (Å)	d_{ee} (Å)
7	10.559	0.082
8	12.075	3.114
9	13.561	6.086
10	15.051	9.066
11	16.562	12.088
12	18.054	15.072

This rationalizes the choice of a minimum axial displacement index of 8. Smaller axial

displacement indexes give very short (or negative) intralayer end-end distances. For example, axial index 7 gives $d_{\mathrm{ee}} = 0.082$ Å which is far below the sum of two sulfur van der Waals radii ($2r_{\mathrm{vdW}}(\mathrm{S}) = 3.60$ Å).[5] Packing structures generated with indexes below 8 are therefore expected to involve severe steric overlap and are thus highly energetically unfavorable.

AA vs AB cohesive energy

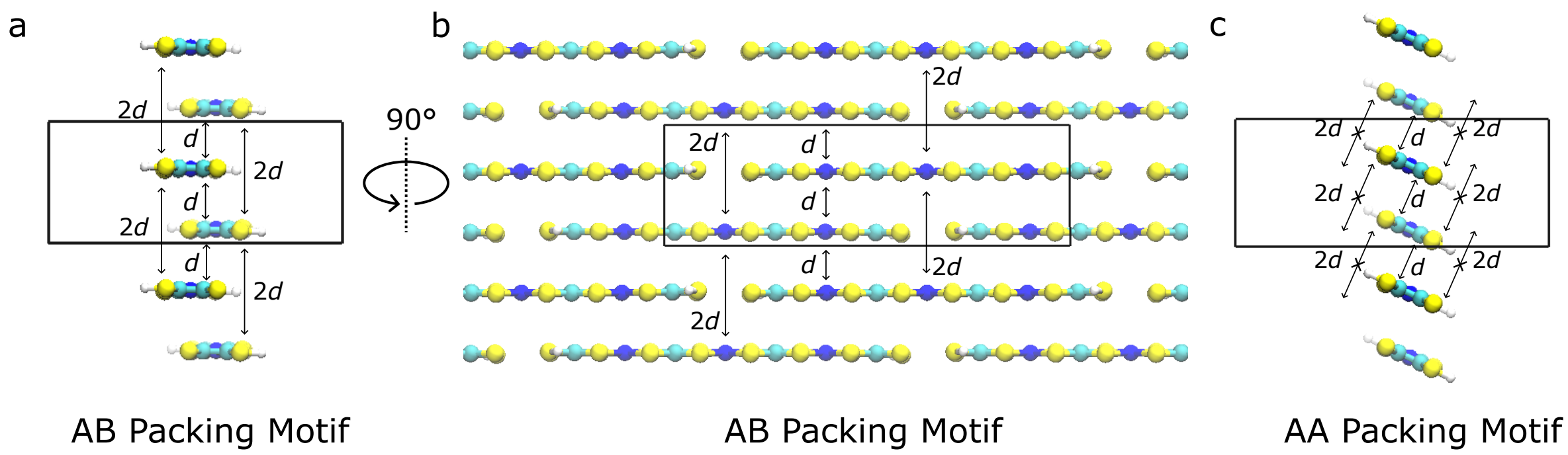

Figure S6: **Next-nearest-neighbor interlayer interactions.** Possible nearest-neighbor (d) and next-nearest-neighbor ($2d$) interlayer interactions are indicated with double-headed arrows for the AB packing motif viewed along the lateral axis (**a**) and axial axis (**b**). Nearest-neighbor interactions (d) are similarly shown for the AA packing motif viewed along the lateral axis (**c**). The absence of next-nearest-neighbor interactions ($2d$) is indicated by crossed double-headed arrows.

Frontier orbitals from POD

Table S3: Electronic coupling values (meV) for hole (h^+) and excess electron (e^-) transport obtained using POD in vacuum (Vac) and condensed phase (PBC), for systems in centrosymmetric (CS) and non-centrosymmetric (NCS) alignments.

Configuration	Vac (h^+)	PBC (h^+)	Vac (e^-)	PBC (e^-)
AB CS Ax8 Lat0	187	149	98.5	184
AB CS Ax8 Lat1	154	125	93	155
AB CS Ax8 Lat2	85.8	81.3	77	103
AB CS Ax8 Lat3	33.3	63.7	64.7	72.1
AB CS Ax8 Lat4	2.64	60.7	62.7	66.1
AB NCS Ax8 Lat0	104	98.4	98.0	181
AB NCS Ax8 Lat1	94.0	85.5	90.1	153.5
AB NCS Ax8 Lat2	77.6	65.9	72.7	101.5
AB NCS Ax8 Lat3	88.1	69.9	58.5	70.6
AB NCS Ax8 Lat4	106	81.1	55.3	63.8

As discussed in the main text, electronic coupling is primarily dictated by orbital overlap. Hence, orbital polarization inherently influences electronic coupling values for both hole and excess electron coupling, with hole coupling being overestimated and excess electron coupling underestimated - this is reflected in Table S3. POD electronic couplings calculated in the condensed phase were done so under 1D periodic boundary conditions, periodic in the axial direction as orbital polarization is primarily along the axial direction (Figure S7). Additionally, extension (in the axial direction) of the primitive unit cell to include 4 monomers was necessary to avoid finite system size errors for electronic coupling calculations under periodic boundary conditions. This also distinguishes between 'left' and 'right' electronic couplings (Figure S8).

Interestingly we observe that for centrosymmetric configurations, 'left' and 'right' electronic coupling values are non-equivalent, with larger differences observed for larger lateral displacements. The same is not observed for non-centrosymmetric configurations, with 'left'/'right' electronic coupling differences typically being smaller than 2 meV. The 'left'/'right' non-equivalence in electronic coupling observed for CS configurations arises due to a 'left'/'right' non-equivalence in overlap environments. Evaluation of the influence of 'left'/'right' non-

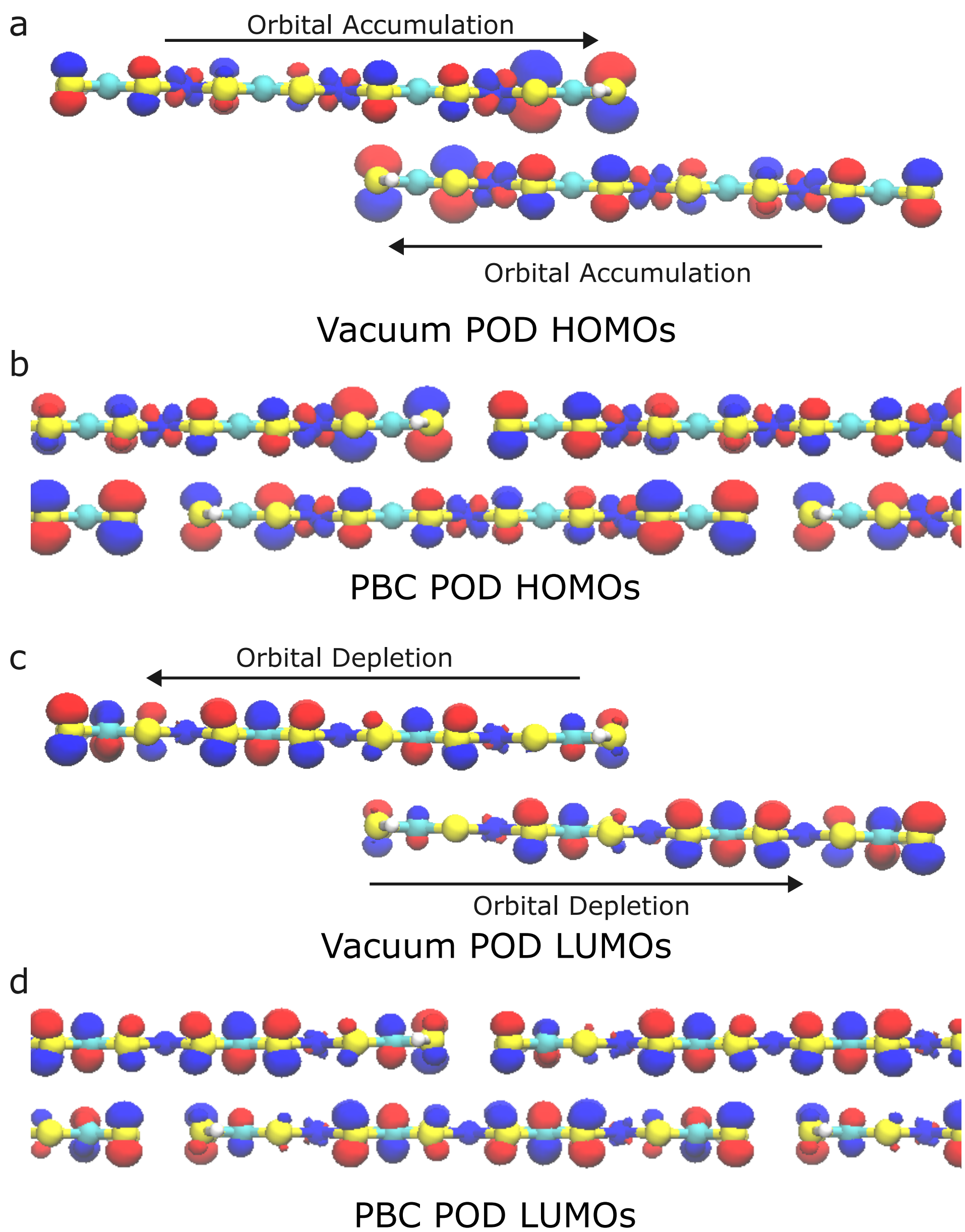

Figure S7: Frontier orbitals obtained from diabatization using the POD method. In **a** and **c** calculations are carried out for a NiBiD dimer in vacuum and the isosurfaces depict the HOMO (**a**) or LUMO (**c**) localized on each of the two monomers forming the dimer as obtained from projection operator-based diabatization (POD). Notice the accumulation (**a**) or depletion (**c**) of orbital amplitudes in the molecular overlap region (i.e. orbital polarization). In **b** and **d** calculations are carried our for NiBiD nanoribbons in PBC and the isosurfaces depict again the POD HOMO (**b**) or POD LUMO (**d**) localized on each molecule of the periodic system. Notice that the localized frontier orbitals in the periodic system show little polarization and resemble closely the frontier orbitals of the monomer in the gas phase, Fig.1a-b, main text.

equivalence on charge transport is outside the current scope of investigation hence, the mean of 'left' and 'right' electronic coupling values are taken for all structures except those that distort.

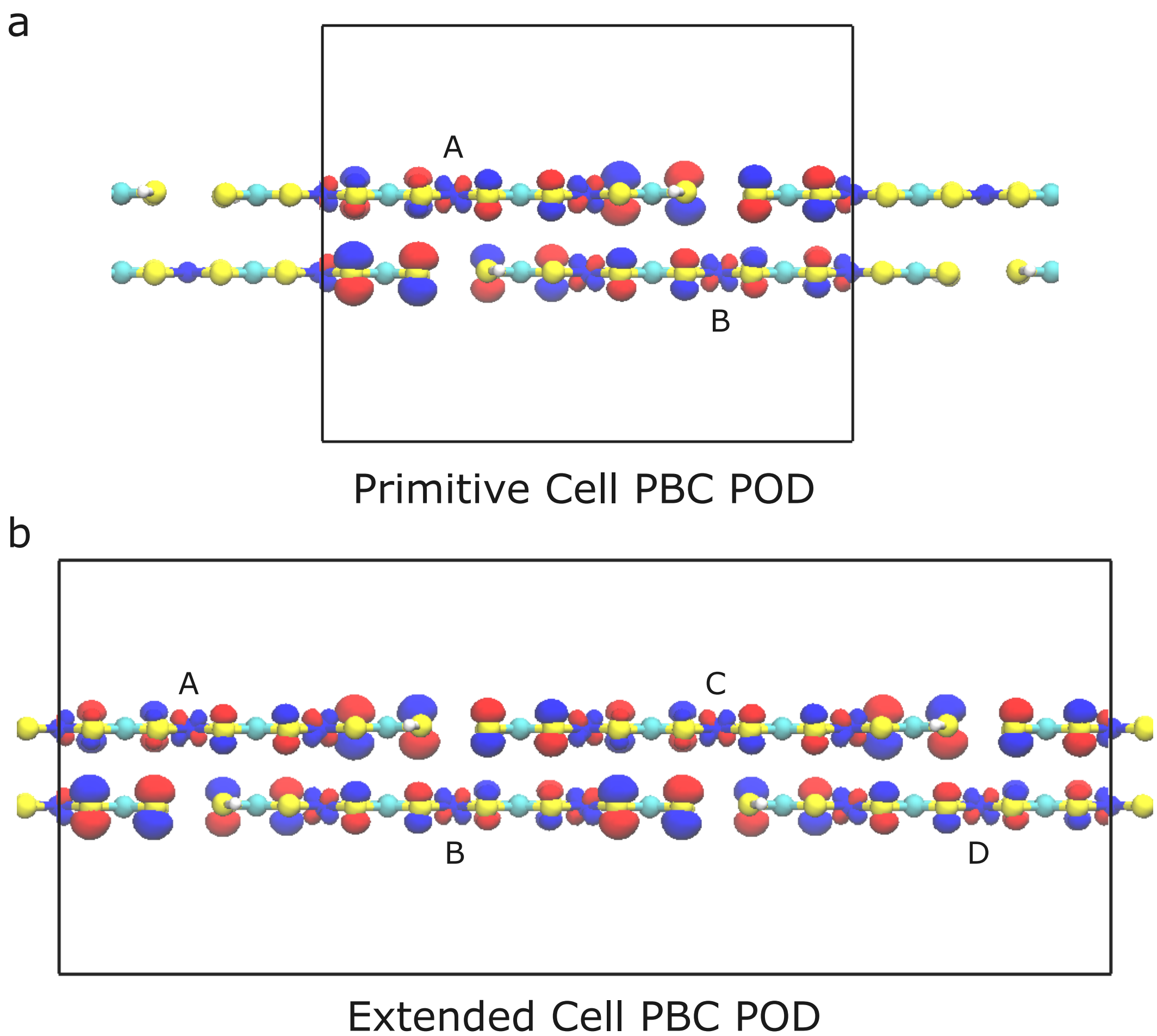

Figure S8: **Choice of supercell for electronic coupling calculations in PBC.** In **a**, the primitive unit cell (2 molecules per cell) is shown together with the POD HOMO orbitals for molecules A and B. Neither orbital is fully represented within the unit cell; instead, the orbitals wrap across the periodic boundaries, leading to double counting of orbital overlap. An axially extended unit cell is shown in **b**, together with the POD orbitals for molecules A-D. In this case, periodic orbital wrapping no longer influences orbital overlap. Additionally, 'left' (orbital B to orbital A, denoted 'L' in Table S4) and 'right' (orbital B to orbital C, denoted 'R' in TableS4) electronic couplings can be distinguished.

Table S4: Electronic coupling values (meV) for hole (h^+) and excess electron (e^-) transport obtained from calculations in PBC using POD. Both non-equivalent coupling values left (L) and right (R) and their mean are provided, see Fig. S8 for definition of L and R.

Configuration	L/R (h^+)	Mean (h^+)	L/R (e^-)	Mean (e^-)
AB CS Ax8 Lat0	130, 168	149	172, 195	184
AB CS Ax8 Lat1	95.5, 154	125	159, 150	155
AB CS Ax8 Lat2	50.6, 112	81.3	115, 90.8	103
AB CS Ax8 Lat3	22.3, 105	63.7	84.9, 59.2	72.1
AB CS Ax8 Lat4	7.44, 114	60.7	78.5, 53.7	66.1
AB NCS Ax8 Lat0	98.9, 97.9	98.4	180, 181	181
AB NCS Ax8 Lat1	86.8, 84.2	85.5	155, 152	154
AB NCS Ax8 Lat2	66.2, 65.5	65.9	103, 100	102
AB NCS Ax8 Lat3	69.2, 70.6	69.9	71.5, 69.7	70.6
AB NCS Ax8 Lat4	81.1, 81.1	81.1	64.1, 63.5	63.8

Non-centrosymmetric plot

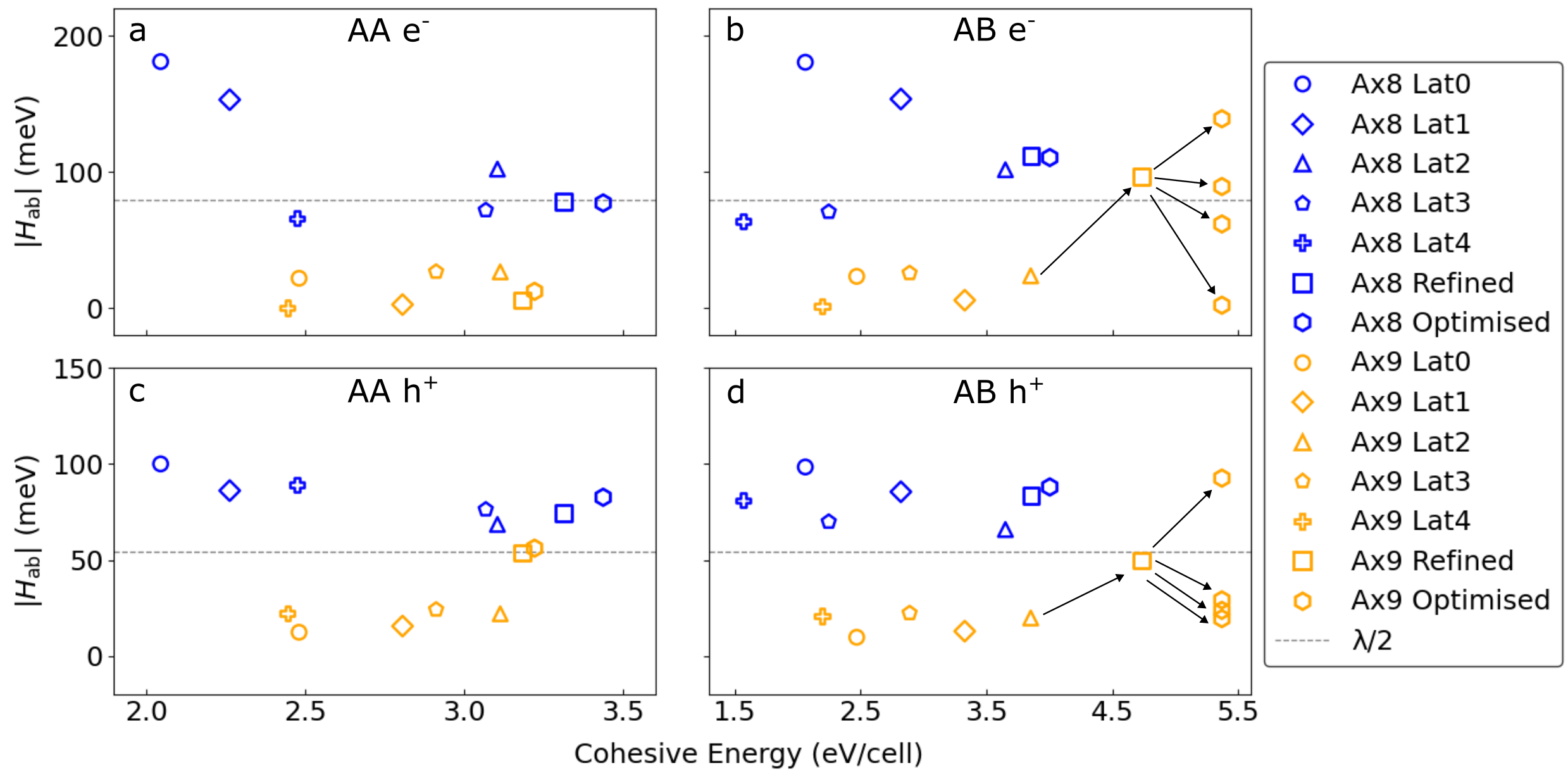

Figure S9: **Electronic coupling versus packing stability for non-centrosymmetric nanoribbon model structures.** Electronic coupling (y axis) and cohesive energy (x axis) are plotted for centrosymmetric packing structures, with color indicating axial displacement index and marker shape indicating lateral displacement index. Refined and optimized structures are shown as squares and hexagons, respectively. AA and AB packing motifs are shown for excess electron coupling in **a** and **b**, respectively, and for hole coupling in **c** and **d**, respectively. Dashed gray lines indicate the $\lambda/2$ threshold, as in Fig. 2. Black arrows in **b** and **d** indicate the progression from 'Coarse' to 'Refined' structures (triangle to square) and from 'Refined' to 'Optimized' structures (square to hexagon) for the highest stability structures. Analogous data for centrosymmetric configurations are shown in Fig. 5 in the main text.

Coupling values

Table S5: Electronic coupling values (meV) for hole (h^+) and excess electron (e^-) transport obtained using condensed phase POD for centrosymmetric (CS) configurations plotted in Fig. 4.

Configuration	h^+	e^-
AA CS Ax8 Lat0	152	184
AA CS Ax8 Lat1	125	154
AA CS Ax8 Lat2	82.7	102
AA CS Ax8 Lat3	70.2	73.0
AA CS Ax8 Lat4	70.5	68.1
AA CS Ax8 Refined	77.3	82.7
AA CS Ax8 Optimized	76.6	81.5
AA CS Ax9 Lat0	49.0	15.7
AA CS Ax9 Lat1	35.0	19.0
AA CS Ax9 Lat2	12.2	31.0
AA CS Ax9 Lat3	12.2	29.3
AA CS Ax9 Lat4	9.40	8.21
AA CS Ax9 Refined	43.0	24.6
AA CS Ax9 Optimized	43.1	22.0
AB CS Ax8 Lat0	149	184
AB CS Ax8 Lat1	125	155
AB CS Ax8 Lat2	81.3	103
AB CS Ax8 Lat3	63.7	72.1
AB CS Ax8 Lat4	60.7	66.1
AB CS Ax8 Refined	71.0	80.3
AB CS Ax8 Optimized	104	115
AB CS Ax9 Lat0	49.2	17.2
AB CS Ax9 Lat1	38.0	12.6
AB CS Ax9 Lat2	17.3	28.4
AB CS Ax9 Lat3	10.1	28.2
AB CS Ax9 Lat4	8.85	9.42
AB CS Ax9 Refined	88.4	107
AB CS Ax9 Optimized	0, 217, 25.1, 60.9	40.8, 178, 44.5, 14.8

Table S6: Electronic coupling values (meV) for hole (h^+) and excess electron (e^-) transport obtained using condensed phase POD for non-centrosymmetric (NCS) configurations plotted in Fig. S9.

Configuration	h^+	e^-
AA NCS Ax8 Lat0	100	181
AA NCS Ax8 Lat1	86.1	153
AA NCS Ax8 Lat2	68.5	102
AA NCS Ax8 Lat3	76.3	71.8
AA NCS Ax8 Lat4	89.2	65.9
AA NCS Ax8 Refined	74.4	78.3
AA NCS Ax8 Optimized	82.7	77.1
AA NCS Ax9 Lat0	12.4	21.8
AA NCS Ax9 Lat1	15.6	2.46
AA NCS Ax9 Lat2	22.0	26.5
AA NCS Ax9 Lat3	24.2	26.6
AA NCS Ax9 Lat4	22.2	0.0250
AA NCS Ax9 Refined	53.8	5.85
AA NCS Ax9 Optimized	56.2	12.2
AB NCS Ax8 Lat0	98.4	181
AB NCS Ax8 Lat1	85.5	154
AB NCS Ax8 Lat2	65.9	102
AB NCS Ax8 Lat3	69.9	70.6
AB NCS Ax8 Lat4	81.1	63.8
AB NCS Ax8 Refined	83.5	112
AB NCS Ax8 Optimized	88.0	111
AB NCS Ax9 Lat0	9.78	23.2
AB NCS Ax9 Lat1	13.0	5.68
AB NCS Ax9 Lat2	19.8	23.6
AB NCS Ax9 Lat3	22.4	25.6
AB NCS Ax9 Lat4	20.9	1.34
AB NCS Ax9 Refined	49.9	96.6
AB NCS Ax9 Optimized	29.2, 18.5, 23.8, 92.6	61.7, 139, 89.2, 2.00

References

(1) Elmaslmane, A. R.; Wetherell, J.; Hodgson, M. J. P.; McKenna, K. P.; Godby, R. W. Accuracy of Electron Densities Obtained via Koopmans-Compliant Hybrid Functionals. *Phys. Rev. Mater.* **2018**, *2*, 040801.

(2) Janak, J. F. Proof that $\partial E/\partial n_i = \epsilon_i$ in Density-Functional Theory. *Phys. Rev. B* **1978**, *18*, 7165–7168.

(3) Koopmans, T. "Uber die Zuordnung von Wellenfunktionen und Eigenwerten zu den einzelnen Elektronen eines Atoms. *Physica* **1934**, *1*, 104–113.

(4) Dudarev, S. L. and Botton, G. A. and Savrasov, S. Y. and Humphreys, C. J. and Sutton, A. P. Electron-energy-loss spectra and the structural stability of nickel oxide: An LSDA+U study. *Phys. Rev. B* **1998**, *57*, 1505–1509.

(5) Bondi, A. van der Waals Volumes and Radii. *J. Phys. Chem.* **1964**, *68*, 441–451.
